# Supplementary material for: Content-rich biological network constructed by mining PubMed abstracts
Source: BMC Bioinformatics. 2004 Oct 8;5:147. doi: 10.1186/1471-2105-5-147 (PMC528731; doi:10.1186/1471-2105-5-147)
Supplement: Additional File 2 — The original results of the above study (non-essential files are deleted to keep the file size under the limit set by BMC bioinformatics). [file 1471-2105-5-147-S2.bz2 › chilibotAdditionalFile2/dip20/299ID7486683E1290/html/RASA1_PDGFRB.html]

 


 **RASA1** and **PDGFRB** 
  
Found 28 abstracts in PubMed, retrieved 20.  
 

 What does Google say? 
 PDF only 
| .edu only 

---

**Interactive relationship** (e.g. stimulation, inhibition, etc)

**Neutral relationship**- RasGAP  [ **RASA1** ] , that responds to activation of PDGFR  [ **PDGFRB** ]  beta but not PDGFR  [ **PDGFRB** ]  alpha, was not phosphorylated on tyrosine in mutant cells upon PDGF treatment.  Ref: 9696037 Oncogene, 1998
- Whereas the PDGFr  [ **PDGFRB** ]  associated with PtdIns 3 kinase, ras GAP  [ **RASA1** ] , GRB 2, and phospholipase C gamma, only GRB 2 and PtdIns 3 kinase associated with IRS 1.  Ref: 7486683 Ann N Y Acad Sci, 1995
- The activated beta PDGFR  [ **PDGFRB** ]  physically associated and phosphorylated signaling molecules such as ras GTPase activating protein  [ **RASA1** ]  GAP  [ **RASA1** ]  and phospholipase C gamma PLC gamma.  Ref: 7489253 Arterioscler Thromb Vasc Biol, 1995
- On ligand induced tyrosine phosphorylation, PDGFR  [ **PDGFRB** ]  associated with phospholipase C PLC gamma 1, Ras GTPase activating protein  [ **RASA1** ]  RasGAP  [ **RASA1** ] , and phosphatidylinositol 3 kinase PI3K but not with the Syp growth factor receptor bound protein 2 Son of Sevenless complex.  Ref: 9612285 Am J Physiol, 1998
- These findings are consistent with the in vitro experiments showign that Syp prefers to dephosphorylate sites on the beta PDGFR  [ **PDGFRB** ] , that are important for binding phosphatidylinositol 3 kinase 740 and Tyr 751 and GAP  [ **RASA1** ]  771.  Ref: 7545675 J Biol Chem, 1995
- In addition, the GTPase activating protein  [ **RASA1** ]  of Ras and phosphatidylinositol 3 kinase were less stably associated with the wild type beta PDGFR  [ **PDGFRB** ]  than with the F1009 receptor.  Ref: 7545675 J Biol Chem, 1995
- We constructed and characterized a series of PDGFR  [ **PDGFRB** ]  mutants which contain binding sites for PI3K as well as one additional protein, either RasGAP  [ **RASA1** ] , SHP 2, or PLC gamma.  Ref: 8816504 Mol Cell Biol, 1996
- Upon binding of platelet derived growth factor PDGF, the PDGF beta receptor PDGFR  [ **PDGFRB** ]  undergoes autophosphorylation on distinct tyrosine residues and binds several SH2 domain containing signal relay enzymes, including phosphatidylinositol 3 kinase PI3K, phospholipase C gamma PLC gamma, the GTPase activating protein  [ **RASA1** ]  of Ras RasGAP  [ **RASA1** ] , and the tyrosine phosphatase SHP 2.  Ref: 8816504 Mol Cell Biol, 1996
- We have found that the F2 mutation has only a modest effect on ligand dependent activation of a chimeric PDGFR  [ **PDGFRB** ]  composed of the extracellular and transmembrane domains of the alphaPDGFR and the cytoplasmic domain of the betaPDGFR by three measures 1 the ability to phosphorylate endogenous and exogenous protein substrates in vitro, 2 phosphorylation of tyrosine 857, and 3 binding of the effector proteins PLCgamma, RasGAP  [ **RASA1** ] , and SHP 2.  Ref: 11281646 Exp Cell Res, 2001
- Our results indicate that a delicate balance of migration promoting phospholipase C gamma and phosphatidylinositol 3 OH kinase and migration suppressing GAP  [ **RASA1** ]  activities are recruited by the PDGFR  [ **PDGFRB** ]  beta to drive chemotaxis towards PDGF BB.  Ref: 8107807 Nature, 1994
- Thus, GAP  [ **RASA1** ]  functions to suppress signal relay by the beta PDGFR  [ **PDGFRB** ] , and it does so by silencing PLC gamma.  Ref: 7760802 Mol Cell Biol, 1995
- Upon stimulation, the tyrosine kinase PDGF receptor beta PDGFR  [ **PDGFRB** ]  beta autophosphorylates and forms a complex that includes SII2 Src homology 2 domain containing proteins such as the phosphatidylinositol specific phospholipase C gamma, Ras GTPase activating protein  [ **RASA1** ]  GAP  [ **RASA1** ] , and phosphatidylinositol 3 OH kinase.  Ref: 8107807 Nature, 1994

**Non-interactive relationship** (e.g. studied together, co-existance, homology, etc.)

- Unlike the PDGFR  [ **PDGFRB** ]  beta subunit, the PDGF activated alpha subunit did not stably associate with the GTPase activating protein  [ **RASA1** ]  of ras rasGAP  [ **RASA1** ] , nor did it mediate tyrosine phosphorylation of rasGAP  [ **RASA1** ] .  Ref: 8290262 Oncogene, 1994
- In this study, we further investigated the role of GAP  [ **RASA1** ]  and Syp in mitogenic signaling by beta PDGFR  [ **PDGFRB** ] .  Ref: 7760802 Mol Cell Biol, 1995
- PDGF stimulated the association of PDGFR  [ **PDGFRB** ]  beta with p85, ras GTPase activating protein  [ **RASA1** ]  and PLC gamma.  Ref: 10697503 Anticancer Res< MedlineTA>Anticancer Res, 1995
- Likewise, phosphorylation and association of GAP  [ **RASA1** ]  and PLC gamma with PDGF BB activated beta PDGFR  [ **PDGFRB** ]  were unaffected.  Ref: 7489253 Arterioscler Thromb Vasc Biol, 1995
- Furthermore, we demonstrate that p62 dok can exert its negative effect on the PDGFR  [ **PDGFRB** ]  MAPK pathway independently of its ability to associate with RasGAP  [ **RASA1** ]  and Nck.  Ref: 11489946 J Exp Med, 2001
- Focusing on the PLC gamma dependent branch of beta PDGFR  [ **PDGFRB** ]  signaling, we constructed a series of mutant beta PDGFRs that contained the binding sites for pairs of the receptor associated proteins PLC gamma and PI3K, PLC gamma and GAP  [ **RASA1** ] , or PLC gamma and Syp.  Ref: 7760802 Mol Cell Biol, 1995
- We conclude that the PDGFR  [ **PDGFRB** ]  alpha subunit does not mediate tyrosine phosphorylation or associate with ras GAP  [ **RASA1** ] ,.  Ref: 8290262 Oncogene, 1994
- Association of PDGFR  [ **PDGFRB** ]  with PLC gamma 1 and RasGAP  [ **RASA1** ]  followed by their tyrosine phosphorylation failed to activate PLC gamma 1, protein kinase C PKC, and Ras.  Ref: 9612285 Am J Physiol, 1998
- SB, in the absence of PDGF BB, caused neither beta PDGFR  [ **PDGFRB** ]  tyrosine phosphorylation nor phosphorylation and association of GAP  [ **RASA1** ]  and PLC gamma with beta PDGFR  [ **PDGFRB** ] .  Ref: 7489253 Arterioscler Thromb Vasc Biol, 1995
